# An Artifact in Intracellular Cytokine Staining for Studying T Cell Responses and Its Alleviation

**DOI:** 10.3389/fimmu.2022.759188

**Published:** 2022-01-21

**Authors:** Zheng Gong, Qing Li, Jiayuan Shi, Guangwen Ren

**Affiliations:** ^1^ The Jackson Laboratory, Bar Harbor, ME, United States; ^2^ The Jackson Laboratory Cancer Center, Bar Harbor, ME, United States

**Keywords:** intracellular cytokine staining, neutrophils, cancer, inflammation, immunosuppression, hydrogen peroxide

## Abstract

Intracellular cytokine staining (ICS) is a widely employed *ex vivo* method for quantitative determination of the activation status of immune cells, most often applied to T cells. ICS test samples are commonly prepared from animal or human tissues as unpurified cell mixtures, and cell-specific cytokine signals are subsequently discriminated by gating strategies using flow cytometry. Here, we show that when ICS samples contain Ly6G^+^ neutrophils, neutrophils are *ex vivo* activated by an ICS reagent – phorbol myristate acetate (PMA) – which leads to hydrogen peroxide (H_2_O_2_) release and death of cytokine-expressing T cells. This artifact is likely to result in overinterpretation of the degree of T cell suppression, misleading immunological research related to cancer, infection, and inflammation. We accordingly devised easily implementable improvements to the ICS method and propose alternative methods for assessing or confirming cellular cytokine expression.

## Introduction

Intracellular cytokine staining (ICS) is a prevailing method for measuring cytokine expression in immune cells at the single cell level, especially T cells (1). The test samples typically undergo a short period (~3-6 hours) of *ex vivo* activation by mitogens or antigens, which elicit primary cytokine production in immune cells mimicking physiological conditions (2). The presence of protein transport inhibitors, such as brefeldin A, limits cytokine localization to the rough endoplasmic reticulum, enabling subsequent immunostaining and intracellular cytokine detection upon sample fixation and permeabilization (1). As ICS permits simultaneous detection of multiple cytokine signals within a single cell, it has been widely adopted in the research fields of infection, inflammation, and cancer for quantitative analysis of immune responses (1).

To better capture the *in vivo* activation status of immune cells, freshly dissociated cells from animal or human crude tissues are commonly used in bulk for direct ICS tests. Identification of the cytokine expression levels in specific cell types is then achieved *via* selective cell gating during flow cytometric analysis (1). We searched research papers published in select leading immunology journals in 2019-2020 and found that crude samples were used more often than purified cell samples in ICS analysis (**Figure S1A**). In particular, in recent COVID-19-related studies, crude clinical samples were predominantly employed for ICS (**Figure S1A**).

In the present work that determines the T cell responses under various inflammatory conditions, we found that when ICS samples contain neutrophils, one of the most abundant immune cells in human and animals (3), neutrophils are simultaneously activated by the T cell activation agent phorbol myristate acetate (PMA) *ex vivo*. The PMA-stimulated neutrophils release hydrogen peroxide (H_2_O_2_), which in turn kills cytokine-expressing T cells during the initial step of ICS. This leads to an artifactual and inaccurate result of apparent robust T cell immunosuppression in crude samples containing neutrophils. We accordingly provided easily executed correction methods for ICS, and complementary methods in assessing and confirming cellular cytokine expression in immune cells.

## Materials and Methods

### Mice

The animal study was reviewed and approved by the Institutional Animal Care and Use Committee at The Jackson Laboratory. C57BL/6J, BALB/cJ, IFNγ-YFP (C.129S4(B6)-*Ifng^tm3.1Lky^*/J), *Nox2* ^-/-^ (B6.129S-*Cybb^tm1Din^*/J), OT-1 (C57BL/6-Tg(TcraTcrb)1100Mjb/J), OT-2 (B6.Cg-Tg(TcraTcrb)425Cbn/J) and humanized CD34^+^ mice were obtained from The Jackson Laboratory (Bar Harbor, ME, USA).

### Tumor Cell Lines

Mouse mammary cancer cell line 4T1 and human mammary cancer cell line MDA-MB-231 were purchased from American Type Culture Collection (ATCC; Manassas, Virginia, USA). The AT3 cell line was provided by S.I. Abrams (Roswell Park Comprehensive Cancer Center). The tumor cells were cultured in DMEM (Gibco, Thermo Fisher Scientific, Inc., Waltham, MA, USA) or RPMI-1640 (Gibco, Thermo Fisher Scientific, Inc., Waltham, MA, USA) supplemented with 10% fetal bovine serum (FBS) (Gemini Bio-Products, West Sacramento, CA, USA), 100 U/mL penicillin, and 100 μg/mL streptomycin (Gibco, Thermo Fisher Scientific, Inc., Waltham, MA, USA). All cells were cultured in a 5% CO_2_ humidified incubator at 37°C. All cell lines used in this study were determined to be negative for *Mycoplasma* prior to experiments. In order to overexpress mouse G-CSF, AT3 cells were infected with *gcsf* (*Csf3*)-expressing lentivirus (the vector was a gift from R.A. Weinberg, Massachusetts Institute of Technology). AT3-*gcsf* construct positive cells were selected with G418 sulfate (Thermo Fisher Scientific, Inc., Waltham, MA, USA).

### *In Vivo* Tumor Implantation

For orthotopic tumor formation, cultured tumor cells (at a density of 2×10^5^ cells) were suspended in 10 μl sterile PBS and injected into the fourth mammary fat pads of female BALB/cJ, IFNγ-YFP, C57BL/6J, *Nox2* ^-/-^ or humanized CD34^+^ mice. Detection of the immune responses, including mouse or human cytokine-expressing T cells, were conducted at the early pre-metastatic stage when primary tumors were palpable (day 12 and 16 for the 4T1 and AT3/AT3-*gcsf* models, and day 30 for the MDA-MB-231 model).

### Tissue and Primary Tumor Dissociation

The lung and primary tumors were collected from euthanized mice and cut into small pieces. Then the tissues were digested with 1 mg/ml collagenase IV (Thermo Fisher Scientific, Inc., Waltham, MA, USA) and 0.1 mg/ml DNase I (Sigma-Aldrich, St. Louis, MO, USA) in RPMI-1640 (10% FBS) medium for 1 hour (tumor samples for 30 minutes) at 37°C. Enzyme activity was neutralized by addition of medium and dissociated tissues were then filtered through 100 µm cell strainers. The spleens were smashed using a syringe and the suspension was filtered with 70 µm cell strainers. The red blood cells were lysed using ACK lysis buffer (Thermo Fisher Scientific, Inc., Waltham, MA, USA) and all the single cells were then passed through 40 µm cell strainers.

### T Cell Isolation and Neutrophil Depletion

Pan T cells were isolated from spleen or lung cells in naïve or tumor-bearing mice using anti-CD90.2 magnetic beads (Miltenyi Biotech, Auburn, CA, USA) according to the manufacturer’s instructions. Neutrophils were depleted with anti-Ly6G MicroBeads (Miltenyi Biotech, Auburn, CA, USA). The purity of T cells and the efficiency of neutrophils depletion were analyzed with flow cytometry, which were above 90% and 99%, respectively.

### Flow Cytometry and Intracellular Cytokine Staining

For cell-surface staining, single cell suspensions were stained with fluorescent antibodies directly conjugated to cell surface markers. The cells were incubated for 30 min at 4°C and DAPI was added to indicate dead cells.

For intracellular cytokine staining, the cells were first incubated for 4 hours with 25 ng/ml PMA (Sigma-Aldrich, St. Louis, MO, USA), 1 µg/ml ionomycin (Sigma-Aldrich, St. Louis, MO, USA), and GolgiPlug (1:1000; BD Biosciences, San Jose, CA, USA) (with or without catalase, 1000 U/ml) in a tissue culture incubator at 37°C. Subsequently, Live/Dead Fixable stain BV510 (1:1000; Thermo Fisher Scientific, Inc., Waltham, MA, USA) was added to exclude dead cells. Surface antibodies were first stained, and cells were permeabilized and fixed using the Cytofix/Cytoperm kit (BD Biosciences, San Jose, CA, USA) according to the manufacturer’s instructions. Then the intracellular proteins were stained with fluorescent antibodies. For the quantification of cell viability in ICS analysis, the αβ T cells or γδ T cells were firstly gated as CD45^+^CD3^+^TCR-β^+^ or CD45^+^CD3^+^TCR-γ/δ^+^, and the viable cells among them were further gated according to negative staining for Live/Dead Fixable stain BV510. Fluorescence intensity was measured on a Symphony A5 (BD Biosciences, San Jose, CA, USA) and data were analyzed by BD FACSDiVa software (version 8) or FlowJo Software (version 10.7.1). Fluorescence-activated cell sorting (FACS) was performed on a FACSAria II (BD Biosciences, San Jose, CA, USA) cell sorter. All antibodies were purchased from Biolegend (San Diego, CA, USA).

To activate T cells with anti-CD3/anti-CD28, the crude lung or spleen samples were prepared from the orthotopic 4T1- or AT3/AT3-*gcsf* tumor bearing mice, and activated by plate-bound anti-CD3 (5 µg/ml; Bio X Cell, Lebanon, NH, USA) and soluble anti-CD28 (1 µg/ml; Bio X Cell, Lebanon, NH, USA) for 24 hours at 37°C. GolgiPlug (1:1000; BD Biosciences, San Jose, CA, USA) was added at the final 4 hours of incubation.

To activate T cells with specific antigen, the OT-1 or OT-2 mice were orthotopically implanted with AT3 or AT3-*gcsf* tumor cells, and at day 16, the crude lung or spleen samples were harvested and activated by OVA_257-264_ peptide (10 µg/ml; *In vivo*Gen, San Diego, CA, USA) or OVA_323-339_ peptide (10 µg/ml; *In vivo*Gen, San Diego, CA, USA), respectively, for 24 hours at 37°C. GolgiPlug (1:1000; BD Biosciences, San Jose, CA, USA) was added at the final 4 hours of incubation.

Mouse T cell panel: CD45-Alexa Flour 700 (Clone: 30-F11); CD3-APC (Clone: 17A2); TCR-β-APC/Fire 750 (Clone: H57-597); TCR-γ/δ-PE (Clone: UC7-13D5); IL-17A-Brilliant Violet 605 (Clone: TC11-18H10.1); IFN-γ-Brilliant Violet 711 (Clone: XMG1.2); IL-2- PE/Cyanine7 (Clone: JES6-5H4); TNF-α-Brilliant Violet 421(Clone: MP6-XT22).

Mouse neutrophil panel: CD45-Alexa Flour 700 (Clone: 30-F11); CD11b-Brilliant Violet 650 (Clone: M1/70); Ly-6C-Brilliant Violet 570 (Clone: HK1.4); Ly-6G-Pacific Blue (Clone: 1A8).

Human T cell panel: CD45-Alexa Flour 700 (Clone: 2D1); CD3-APC (Clone: OKT3); IL-17A-Brilliant Violet 605 (Clone: BL168); IFN-γ-Brilliant Violet 711 (Clone: 4S.B3); IL-2- PE/Cyanine7 (Clone: MQ1-17H12); TNF-α-Brilliant Violet 421(Clone: MAb11).

Human neutrophil panel: CD45-Alexa Flour 700 (Clone: 2D1); CD33-PE/Cyanine5 (Clone: WM53); CD15-Brilliant Violet 650 (Clone: W6D3); CD66b-PerCP/Cyanine5.5 (Clone: G10F5).

### RNA Extraction and qRT-PCR

Total RNA was isolated using the Direct-zol RNA Miniprep Plus Kit (Zymo-Research, Irvine, CA, USA) according to the manufacturer’s instructions. cDNA was synthesized from RNA with the High-Capacity cDNA Reverse Transcription Kit (Thermo Fisher Scientific, Inc., Waltham, MA, USA). qRT-PCR was carried out on the ViiA 7 Real-Time PCR System (Thermo Fisher Scientific, Inc., Waltham, MA, USA) by using PowerUp SYBR Green PCR Master Mix (Thermo Fisher Scientific, Inc., Waltham, MA, USA). Relative mRNA expression was calculated using the comparative CT method (ΔΔCt) normalized to housekeeping gene *Rps18*.

Primer sequences were listed below:

*Rps18* forward, 5’- GGAGAACTCACGGAGGATGA -3’,
*Rps18* reverse, 5’- CCAGTGGTCTTGGTGTGCTG -3’
*Il17a* forward, 5’- CTCCAGAAGGCCCTCAGACTAC -3’,
*Il17a* reverse, 5’- AGCTTTCCCTCCGCATTGACACAG -3’
*Ifng* forward, 5’- GGCCATCAGCAACAACATAAGCGT -3’,
*Ifng* reverse, 5’- TGGGTTGTTGACCTCAAACTTGGC -3’
*Prdx1* forward, 5’- GTTGGCCGCTCTGTGGATGAGAT -3’,
*Prdx1* reverse, 5’- ATCACTGCCAGGTTTCCAGCCAGC -3’
*Prdx2* forward, 5’- GTTCTCCGGCCTAGGGCTCTCTC -3’,
*Prdx2* reverse, 5’- GCCGGAGGCCATGACTGCGTG -3’
*Txn2* forward, 5’- CGACCTTTAACGTCCAGGATG -3’,
*Txn2* reverse, 5’- ACTGTGCATGAAAGTCCACAAC -3’
*Mdh2* forward, 5’- TGACCTGTTCAACACCAACG -3’,
*Mdh2* reverse, 5’- GATGGGGATGGTGGAGTTC -3’
*Sod1* forward, 5’- TACTGATGGACGTGGAACCC -3’,
*Sod1* reverse, 5’- GAACCATCCACTTCGAGCA -3’
*Nfe2l2* forward, 5’- GCAGCCATGACTGATTTAAGC -3’,
*Nfe2l2* reverse, 5’- CAGCCAGCTGCTTGTTTTC -3’
*Gclm* forward, 5’- AGGAGCTTCGGGACTGTATCC -3’,
*Gclm* reverse, 5’- GGGACATGGTGCATTCCAAAA -3’
*Pgd* forward, 5’- ATGGCCCAAGCTGACATTG -3’,
*Pgd* reverse, 5’- GCACAGACCACAAATCCATGAT -3’
*Gpx1* forward, 5’- CAATGTAAAATTGGGCTCGAA -3’,
*Gpx1* reverse, 5’- GTTTCCCGTGCAATCAGTTC -3’
*Gpx4* forward, 5’- TAAGAACGGCTGCGTGGT -3’,
*Gpx4* reverse, 5’- GTAGGGGCACACACTTGTAGG -3

### Quantification of Hydrogen Peroxide

Hydrogen peroxide (H_2_O_2_) quantification was measured using an Amplex Red hydrogen peroxide/peroxidase assay kit (Invitrogen, Thermo Fisher Scientific, Inc., Waltham, MA, USA) according to the manufacturer’s instructions. Briefly, the Amplex Red reagent/HRP working solution was added to each microplate well, and the suspension of isolated neutrophils or crude lung cells (2 × 10^4^ cells) was added to the reaction mixture. Catalase (1000 U/ml) was mixed together with the suspension of cells. Reactions were incubated at room temperature for 30 minutes and fluorescence was measured using a microplate reader (SpectraMax i3, Molecular Devices, Sunnyvale, CA, USA) with excitation at 540 nm and emission at 590 nm.

### H_2_O_2_ Susceptibility Assay

Lung T cells were isolated from IFNγ-eYFP mice and stimulated with 25 ng/ml PMA and 1 µg/ml ionomycin in the presence of GolgiPlug (1:1000) for 4 hours at 37°C. Then the activated T cells were incubated with different concentrations of H_2_O_2_ at 37°C for 2 hours. Viable IFNγ^-^ or IFNγ^+^ T cells were indicated as propidium iodide (PI) negative, and the absolute cell numbers were calculated using Precision Count Beads (Biolegend, San Diego, CA, USA) following the manufacturer’s instructions. The fold change in viable cell numbers was normalized to the control group.

### Illustration Tool

The schematic images are created with Biorender.com.

### Statistical Analysis

Data are presented as mean ± s.e.m. Statistical analyses were performed using GraphPad Prism version 8 software. We carried out unpaired two-tailed Student’s *t*-test to compare two groups, and one-way ANOVA with Tukey’s test to compare the variance in three or more groups with one independent factor. When there were effects of two factors on a dependent variable, two-way ANOVA with Šidák’s multiple comparisons test was used. Statistical significance is indicated as ∗ *P* < 0.05, ∗∗ *P* < 0.01, ∗∗∗ *P* < 0.001, ∗∗∗∗ *P* < 0.0001, or NS (not significant).

## Results

### Contradictory Results Were Obtained in Evaluating T Cell Responses by ICS and Other Cytokine Detection Methods in the Mouse Model of Breast Cancer

**Figure 1:**
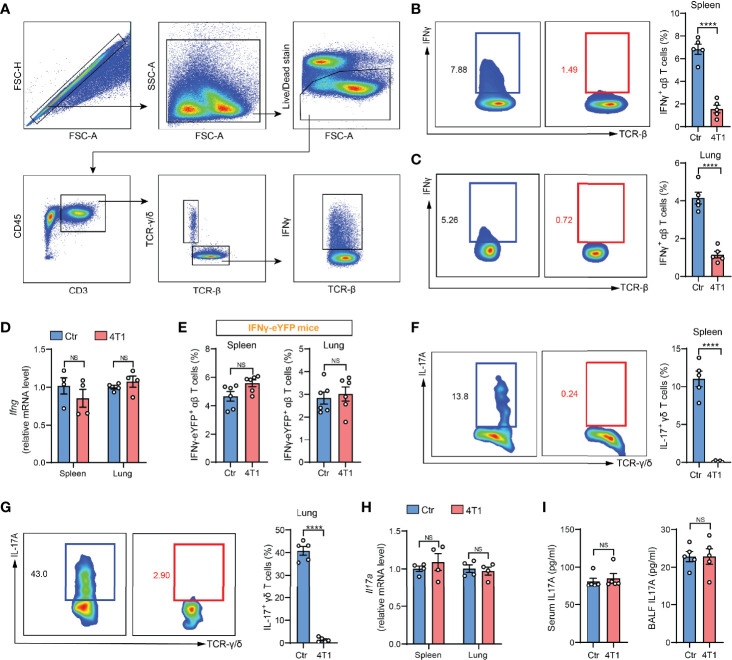
A discrepancy was found between ICS and other cytokine detection methods in assessing T cell responses in the mouse model of breast cancer. **(A)** Gating strategy for spleen IFNγ^+^ αβ T cells in flow cytometric analysis. Arrows indicate the parent population that the subsequent plots are gated on. **(B, C)** ICS analysis of the frequencies of IFNγ^+^ αβ T cells in crude spleen **(B)** or lung **(C)** samples from naïve control (Ctr) or 4T1 tumor-bearing mice (n=5 per group). **(D)** Relative mRNA levels of *Ifng* in purified spleen or lung αβ T cells from control or 4T1 tumor-bearing mice (n=4 per group). **(E)** The frequencies of spleen or lung IFNγ-eYFP^+^ αβ T cells in control or 4T1 tumor-bearing mice (n=6 per group) were quantified by living cell immunostaining and flow cytometry. **(F, G)** ICS analysis of the frequencies of IL-17^+^ γδ T cells in crude spleen **(F)** or lung **(G)** samples from control or 4T1 tumor-bearing mice (n=5 per group). **(H)** Relative mRNA levels of *Il17a* in purified spleen or lung γδ T cells from control or 4T1 tumor-bearing mice (n=4 per group). **(I)** IL-17A levels in serum (*left*) or lung BALF (*right*) from control or 4T1 tumor-bearing mice (n=5 per group) were measured by ELISA. Values of n represent biologically independent animals. Data are mean ± s.e.m. *P* values were calculated using unpaired t-test. ****P < 0.0001. NS, not significant.

In our recent work, we performed ICS to determine IFNγ expression, a common indicator of T cell responses (4), in crude spleen and lung tissue-dissociated cells as a means of assessing cancer-associated systemic immunosuppression at the early tumor progression stage. Using the mouse 4T1 orthotopic breast cancer model, a striking drop in IFNγ^+^ T cells was detected in both spleen and lungs of tumor-bearing mice (**Figures 1A–C**), which suggested tumor-elicited systemic immunosuppression. Surprisingly, this drastic tumor-associated T cell change was not detected when IFNγ expression was measured by quantitative PCR (qPCR) in purified T cells (**Figure 1D**). To untangle these paradoxical results from ICS and qPCR assays, we next leveraged IFNγ-eYFP reporter mice (5), in which IFNγ expression can be quantified by living cell immunostaining followed by flow cytometry instead of by ICS. The results were highly consistent with the qPCR assay, showing that the percentages of IFNγ-eYFP^+^ T cells did not undergo changes with 4T1 tumor growth in either spleen or lungs (**Figure 1E**). These data indicated that contradictory conclusions can be drawn when testing cellular cytokine expression using different methods, and particularly, that there might be defects associated with ICS.

To tease out whether the discrepancy is specific for IFNγ, we next tested another widely studied inflammatory cytokine, IL-17, which is mainly produced by γδ-T cells in various types of inflammatory diseases (6, 7). Using the same 4T1 model, a remarkable reduction in the percentages of IL-17^+^ γδ-T cells was found based on ICS analysis (**Figures 1F, G**), which was again not detected by qPCR in purified spleen or lung-derived γδ-T cells (**Figure 1H**), or by enzyme-linked immunosorbent assay (ELISA) in either serum or bronchoalveolar lavage fluid (BALF) (**Figure 1I**). The recurrent discord between ICS and other cytokine-determining approaches prompted us to reevaluate the application of ICS in assessing T cell cytokine expression using crude samples.

### Host Neutrophilia Contributes to the Artifact Associated With Crude Tissue Sample ICS Analysis in Tumor-Bearing Conditions

**Figure 2:**
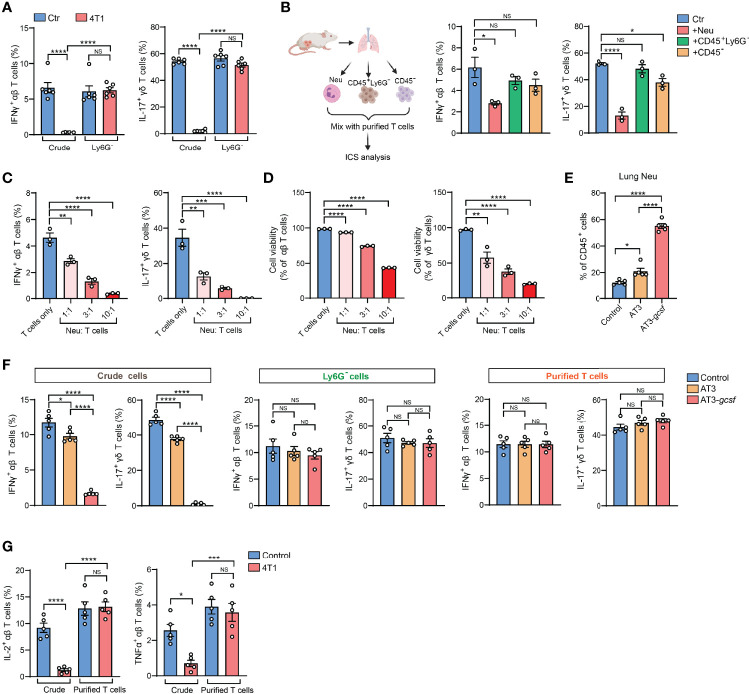
Presence of neutrophils in crude tissue samples accounts for the artifactual results of T cell immunosuppression in tumor-bearing conditions. **(A)** ICS analysis of the frequencies of lung IFNγ^+^ αβ T cells (*left*) or IL-17^+^ γδ T cells (*right*) from control or 4T1 tumor-bearing mice (n=6 per group) with or without neutrophil depletion. **(B)** Purified naïve mice-derived lung T cells were mixed with different types of lung tissue cells (Ly6G^+^ neutrophils, CD45^+^Ly6G^-^ non-neutrophil leukocytes, or CD45^-^ non-leukocyte stromal cells) derived from 4T1 tumor-bearing mice, and the frequencies of IFNγ^+^ αβ T cells or IL-17^+^ γδ T cells were quantified by ICS. **(C, D)** Purified naïve mice-derived lung T cells and 4T1 tumor-bearing mice-derived lung neutrophils were mixed together at the indicated ratios and subjected to ICS analysis. The frequencies of IFNγ^+^ αβ T cells or IL-17^+^ γδ T cells **(C)** were quantified, and the T cell viabilities **(D)** were determined by Live/Dead Fixable staining. **(E)** The percentages of lung neutrophils were compared among control, AT3- and AT3-*gcsf* tumor-bearing mice (n=5 mice per group). **(F)** ICS analysis of the frequencies of lung IFNγ^+^ αβ T cells or IL-17^+^ γδ T cells in crude samples (*left*), neutrophil-depleted samples (Ly6G^-^ cells; *middle*), and purified T cell samples (*right*) in the AT3/AT3-*g-csf* models (n=5 mice per group). **(G)** ICS analysis of the frequencies of lung IL-2^+^ αβ T cells (*left*) or TNFα^+^ αβ T cells (*right*) in crude samples or in purified T cell samples from control or 4T1 tumor-bearing mice (n=5 per group). Values of n represent biologically independent animals. Data are mean ± s.e.m. *P* values were calculated using one-way ANOVA **(A–G)**. *P < 0.05; **P < 0.01; ***P < 0.001; ****P < 0.0001. NS, not significant.

The 4T1 breast tumor model is well known for induction of a profound host inflammation, characterized by a large expansion of neutrophils (neutrophilia) (**Figures S1B, C**), due to tumor-secreted hematopoietic growth factors (8). We thus speculated that neutrophils, a type of innate immune cell possessing non-specific cytotoxic effects (9), present in the crude tissue samples could react with T cells during the *ex vivo* activation process. Indeed, the IFNγ and IL-17 suppression in T cells observed by crude cell ICS analysis was completely undetected by ICS when Ly6G^+^ neutrophils were depleted from the crude samples (**Figure 2A**). Thus, the presence of neutrophils in ICS samples likely accounts for the artifact of T cell cytokine suppression.

To test the possibility that Ly6G^+^ neutrophils directly interfere with T cells during the ICS procedure, crude lung samples freshly prepared from 4T1 tumor-bearing mice were separated into Ly6G^+^ neutrophils (Neu), CD45^+^ Ly6G^-^ leukocytes and non-leukocyte stromal cells (CD45^-^). These different cell subsets were then individually mixed with purified T cells and subjected to ICS analysis (**Figure 2B**, *left*). As expected, incubation with Ly6G^+^ neutrophils, but not CD45^+^ Ly6G^-^ leukocytes or CD45^-^ stromal cells, led to a prominent reduction of IFNγ^+^ and IL-17^+^ T cells (**Figure 2B**, *middle* and *right*). This *ex vivo* Ly6G^+^ neutrophil-mediated cytokine-expressing T cell suppression acted in a dose-dependent manner (*i.e.*, ratio of neutrophils to T cells) (**Figure 2C**), and was likely due to direct T cell killing (**Figures 2D** and **S1D**). Therefore, during crude tissue sample ICS analysis, neutrophils existed in the samples will react with T cells during their *ex vivo* co-incubation resulting in an artifactual T cell suppression.

To validate this neutrophil-mediated artifact and to determine whether it is specific to the 4T1 tumor model, we next analyzed the AT3 and AT3-*gcsf* orthotopic breast tumor models. While AT3 induces marginal host neutrophilia, the AT3-*gcsf* line, which was constructed to overexpress a neutrophil growth factor – granulocyte-colony stimulating factor (G-CSF) (10) – stimulates potent neutrophilia similar to the 4T1 model (**Figures 2E** and **S1E**). By comparing the AT3-*gcsf* and AT3 models, we were able to further confirm whether host neutrophilia causes the ICS-associated artifact. Using crude lung tissue samples, we expectedly observed a pronounced reduction of IFNγ^+^ and IL-17^+^ T cells in the AT3-*gcsf* tumor-bearing mice compared to the AT3-bearing or naïve control mice, by ICS (**Figure 2F**, *left*). However, such a host neutrophilia-associated T cell “suppression” was not detected by ICS analysis when using crude tissue samples depleted of Ly6G^+^ neutrophils, or using purified T cells (**Figure 2F**, *middle* and *right*, and **Figure S2**). These results clearly indicated that the ICS artifact in crude tissue sample analysis occurs when the host develops neutrophilia.

In addition to IFNγ and IL-17, this Ly6G^+^ neutrophil-induced artifactual effect was also found in ICS analysis of other T cell cytokines such as IL-2 and TNFα using crude tissue samples (**Figure 2G**). Moreover, the artifact similarly occurred in non-breast cancer models such as mouse LLC lung carcinoma and MC38 colon adenocarcinoma (**Figure S3**), both of which induce host neutrophilia (**Figure S4**). Taken together, we conclude that host neutrophilia is a primary, if not exclusive, contributor to the artifact associated with crude tissue sample ICS analysis, and this issue can be resolved by Ly6G^+^ neutrophil depletion or T cell purification.

### Hydrogen Peroxide (H_2_O_2_) Is a Primary Mediator of the Neutrophil-Induced Artifact in ICS Analysis

**Figure 3:**
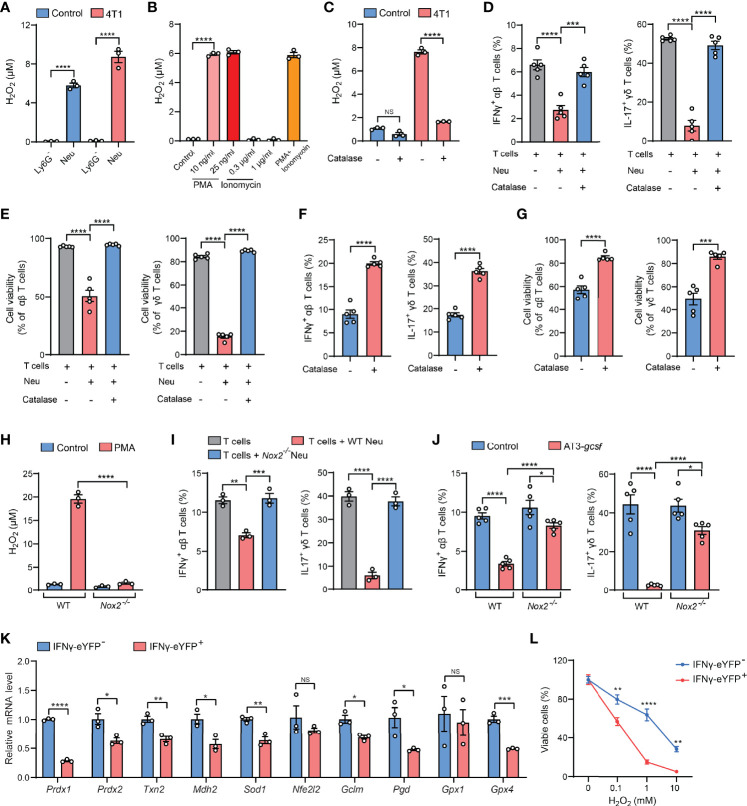
H_2_O_2_ is a key mediator of the neutrophil-induced artifact in ICS analysis. **(A)** H_2_O_2_ levels in isolated Ly6G^-^ or Ly6G^+^ lung tissue cells from control or 4T1 tumor-bearing mice (n=3 per group) were measured after ICS stimulation for 30 minutes at room temperature. **(B)** H_2_O_2_ levels in lung neutrophils isolated from 4T1 tumor-bearing mice (n=3) were measured after incubation with the indicated concentrations of PMA or ionomycin or cotreatment with PMA (25 ng/ml) + ionomycin (1 µg/ml) for 30 minutes at room temperature. **(C)** H_2_O_2_ levels in crude lung samples derived from control or 4T1 tumor-bearing mice were measured after ICS stimulation for 30 minutes at room temperature in the absence or presence of catalase (1000 U/ml). **(D, E)** Purified naïve mice-derived lung T cells and 4T1 tumor-bearing mice-derived lung neutrophils were mixed together in the absence or presence of catalase (1000 U/ml), and then subjected to ICS analysis. The frequencies of IFNγ^+^ αβ T cells or IL-17^+^ γδ T cells **(D)** were quantified, and the T cell viabilities **(E)** were determined by Live/Dead Fixable staining. **(F, G)** Crude 4T1 tumor samples (n=5 per group) were subjected to ICS analysis in the absence or presence of catalase (1000 U/ml). The frequencies of IFNγ^+^ αβ T cells or IL-17^+^ γδ T cells **(F)** were quantified, and the T cell viabilities **(G)** were determined by Live/Dead Fixable staining. **(H)** H_2_O_2_ levels in isolated Ly6G^+^ lung tissue cells from WT or *Nox2* ^-/-^ AT3-*gcsf* tumor-bearing mice were measured after PMA (25 ng/ml) stimulation for 30 minutes at room temperature. **(I)** Purified naïve mice-derived lung T cells were mixed with lung neutrophils derived from WT or *Nox2* ^-/-^ AT3-*gcsf* tumor-bearing mice, and the frequencies of IFNγ^+^ αβ T cells or IL-17^+^ γδ T cells were quantified by ICS (n=3). **(J)** ICS analysis of the frequencies of IFNγ^+^ αβ T cells or IL-17^+^ γδ T cells in crude lung samples from WT or *Nox2* ^-/-^ AT3-*gcsf* tumor-bearing mice (n=5 per group). **(K)** Relative mRNA levels of the indicated antioxidant genes were compared between IFNγ^-^ and IFNγ^+^ lung αβ T cells isolated from IFNγ-eYFP mice (n=5). **(L)** Susceptibility of IFNγ^-^ and IFNγ^+^ lung αβ T cells, isolated from IFNγ-eYFP mice (n=3), to the indicated concentration of H_2_O_2_ for 2 hours. Values of n represent biologically independent animals. Data are mean ± s.e.m. *P* values were calculated using unpaired *t*-test **(F, G, K)** or one-way ANOVA **(A-E, H–J)** or two-way ANOVA **(L)**. *P < 0.05; **P < 0.01; ***P < 0.001; ****P < 0.0001. NS, not significant.

Serving as the essential part of the innate immunity, neutrophils represent one of the most abundant immune cells in animals and humans and play decisive roles in cancer, inflammation and infection (11). At the pathological sites, neutrophils are activated to exert their effector functions such as phagocytosis, degranulation, formation of neutrophil extracellular traps (NETs), and release of reactive oxygen species (ROS) (12). Among these effects, ROS are cytotoxic to both pathogens and the host cells, which partially accounts for the reported dual beneficial and potentially detrimental roles of neutrophils in host defense, tissue damage, and inflammatory diseases (13, 14). We then suspected that the cytotoxic ROS from neutrophils could mediate the killing of cytokine-expressing T cells during the *ex vivo* ICS procedure. By comparing the production of the major ROS component – H_2_O_2_ – in purified Ly6G^+^ neutrophils and Ly6G^+^ neutrophil-depleted lung tissue cells that were both processed by ICS, we found that Ly6G^+^ neutrophils were indeed the primary source of H_2_O_2_ in both naïve and tumor-bearing conditions (**Figure 3A**). Surprisingly, Ly6G^+^ neutrophils that did not undergo the ICS procedure only produce a very low level of H_2_O_2_ (**Figure 3B**, *control* group), suggesting that certain ICS reagents may induce *ex vivo* Ly6G^+^ neutrophil activation to produce H_2_O_2_.

In ICS analysis, immune cells usually need to be activated by mitogens or antigens to mount cytokine responses prior to immunostaining (2). For T cells, the commonly used activation agents are phorbol 12-myristate 13-acetate (PMA) and ionomycin, which are a protein kinase C activator and a calcium ionophore, respectively (15). Although both PMA and ionomycin have been reported to stimulate NET formation and neutrophil apoptosis (16, 17), we found that only PMA but not ionomycin was able to induce H_2_O_2_ production in Ly6G^+^ neutrophils at the same or less concentrations as used in ICS (**Figure 3B**). These results indicated that PMA is a primary ICS agent that activates Ly6G^+^ neutrophils to release cytotoxic H_2_O_2_.

We next attempted to abrogate H_2_O_2_ as a strategy for resolving the PMA-related ICS artifact. To this end, catalase, an antioxidant enzyme with a high capacity to rapidly catalyze H_2_O_2_ decomposition (18), was tested. As expected, H_2_O_2_ levels was substantially reduced by catalase in crude lung samples isolated from tumor-bearing mice (**Figure 3C**). Consequently, addition of catalase in the neutrophil: T cell co-cultures (**Figures 3D, E**), or in the crude tissue samples (**Figure S5A**), significantly mitigated the ICS-associated artifact of T cell suppression (killing). Using catalase addition as a solution to ICS artifact was also found to be effective for intratumoral T cell analysis in crude tumor samples (**Figures 3F, G**), a widely employed measurement to evaluate immunosuppression within the tumor microenvironment. To further affirm the role of H_2_O_2_ in ICS-associated artifact, we utilized *Nox2* ^-/-^ mice which lack the expression of NADPH oxidase 2, a key enzyme in H_2_O_2_ generation (19). Of note, *Nox2* deficiency caused a nearly abolished H_2_O_2_ production in PMA-stimulated Ly6G^+^ neutrophils (**Figure 3H**). In line with this H_2_O_2_ change, in the neutrophil-T cell co-culture system, neutrophil-mediated suppression of IFNγ^+^ and IL-17^+^ T cells was largely reversed by *Nox2* deficiency in Ly6G^+^ neutrophils, as measured by ICS (**Figure 3I**). Furthermore, the artifactual T cell suppression during ICS analysis of crude tissue (lung) samples was significantly mitigated by the host *Nox2* deficiency in the AT3-*gcsf* tumor model (**Figure 3J**). Taken together, H_2_O_2_ was revealed as the key mediator of neutrophil-induced artifact in ICS analysis, and addition of catalase in the ICS procedure was effective in alleviating the H_2_O_2_-associated artifact, representing an improvement to the ICS method when using crude tissue samples containing neutrophils.

Although the mechanisms underlying H_2_O_2_/ROS-mediated killing of cytokine-expressing T cells [representing the activated T cells (20)] need to be further determined, it has been previously reported that IL-17^+^ γδ T cells are susceptible to ROS in the tumor microenvironment due to their low-level expression of the antioxidant glutathione (21). Using the IFNγ-eYFP reporter mice, we found that the IFNγ^+^ T cells similarly express lower levels of a series of antioxidant genes and are more sensitive to H_2_O_2_-induced cell killing, in comparison to their IFNγ^-^ counterparts (**Figures 3K, L**). Therefore, the selective killing of cytokine-expressing T cells by H_2_O_2_/ROS is likely due to their low expression of antioxidants. In addition, H_2_O_2_/ROS was known to mediate NETs formation in neutrophils upon PMA stimulation (22–24), and T cell functions have also been reported to be altered by NETs (25–27). Future efforts need to be made to characterize whether and how NETs play a role in H_2_O_2_/ROS-mediated *ex vivo* T cell modulation in ICS analysis of neutrophil-containing samples.

### The Neutrophil-Induced Artifact Occurs in Other Host Neutrophilia Conditions Than Tumor Models

**Figure 4:**
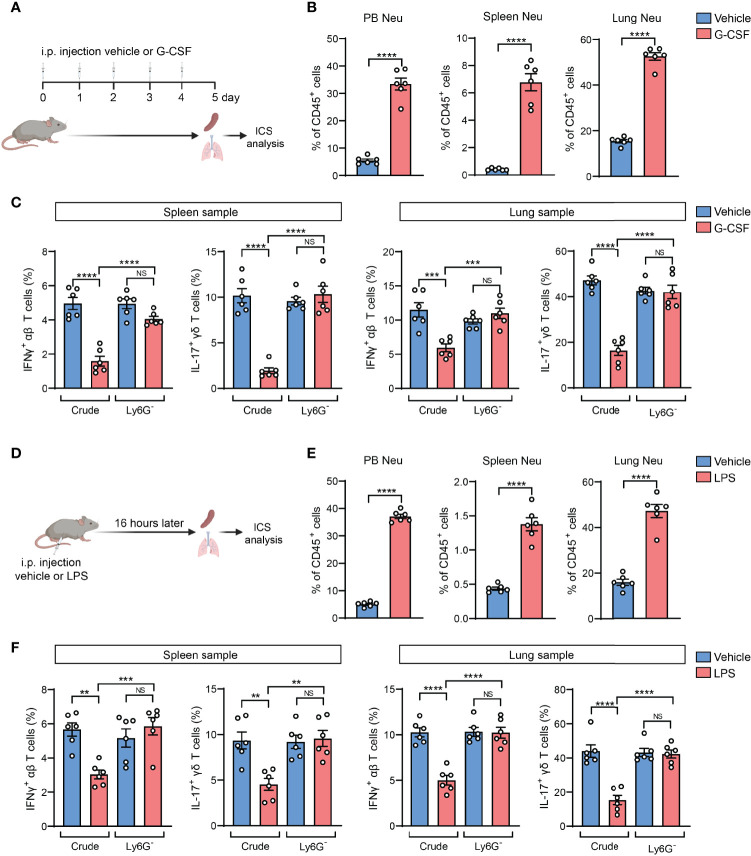
The neutrophil-induced artifact occurs in other host neutrophilia conditions than tumor models. **(A–C)**, As depicted in **(A)** the mice were received i.p. injection of recombinant mouse G-CSF (0.125 mg/kg) or vehicle for 5 consecutive days. The percentages of neutrophils (CD45^+^CD11b^+^Ly6G^+^) in peripheral blood (PB), spleen or lungs were quantified **(B)**. The frequencies of IFNγ^+^ αβ T cells or IL-17^+^ γδ T cells in spleen or lung samples (n=6 per group) with or without neutrophil depletion, were detected ICS and flow cytometry **(C)**. **(D–F)**, As depicted in **(D)** the mice were received i.p. injection of LPS (2.5 mg/kg) or vehicle. 16 hours later, the percentages of neutrophils in PB, spleen or lung were quantified **(E)**. The frequencies of IFNγ^+^ αβ T cells or IL-17^+^ γδ T cells in spleen or lung samples (n=6 per group) with or without neutrophil depletion, were detected by ICS and flow cytometry **(F)**. Values of n represent biologically independent animals. Data are mean ± s.e.m. *P* values were calculated using unpaired *t*-test **(B**, **E)** or one-way ANOVA **(C**, **F)**. **P < 0.01; ***P < 0.001; ****P < 0.0001. NS, not significant.

Based on above findings derived from tumor models, we next asked whether the neutrophil-induced ICS artifact is generalized to other host neutrophilia conditions than tumor models. To this end, we included two experimental models by exogenous injection of G-CSF, the neutrophil-specific growth factor, or exogenous injection of lipopolysaccharide (LPS) which stimulates systemic inflammatory responses (28). Upon G-CSF administration, mice expectedly develop neutrophilia, marked by a striking elevation of Ly6G^+^ neutrophils in different organs and blood circulation (**Figures 4A, B**). Using ICS analysis of the crude spleen and lung tissue samples, we again detected the artifactual suppression of IFNγ^+^ and IL-17^+^ T cells in G-CSF-injected mice, which was not observed using the same crude samples depleted of Ly6G^+^ neutrophils (**Figure 4C**). Similarly, host neutrophilia was remarkably induced in the LPS model (**Figures 4D, E**), and the presence or absence of Ly6G^+^ neutrophils in the crude spleen and lung tissue samples again determined the occurrence of T cell “suppression” or not as analyzed by ICS (**Figure 4F**). Hence, Ly6G^+^ neutrophil-associated ICS artifact occurs at different pathological contexts in which host neutrophilia was induced.

### The ICS-Associated Artifact Occurs in Human Immune Cell Analyses

**Figure 5:**
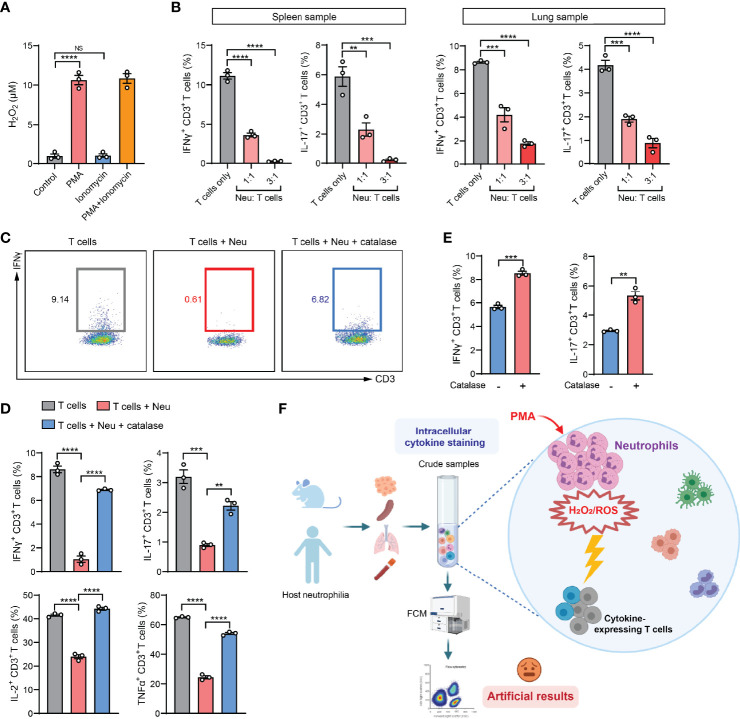
The ICS-associated artifact occurs in human immune cell analyses. **(A)** H_2_O_2_ levels in human neutrophils isolated from lung tissues of MDA-MB-231 tumor-bearing Hu-CD34^+^ mice (n=3) were measured after incubation with PMA (25 ng/ml) or ionomycin (1 µg/ml) or co-treatment with PMA (25 ng/ml) + ionomycin (1 µg/ml) for 30 minutes at room temperature. **(B)** Human T cells and neutrophils were isolated from spleen or lung tissues of MDA-MB-231 tumor-bearing Hu-CD34^+^ mice (n=3), mixed together at the indicated ratios and then subjected to ICS analysis. The frequencies of IFNγ^+^ CD3^+^ T cells or IL-17^+^ CD3^+^ T cells were quantified. **(C, D)** Human T cells and neutrophils were isolated from lung tissues of MDA-MB-231 tumor-bearing Hu-CD34^+^ mice (n=3), mixed together in the absence or presence of catalase (1000 U/ml), and then subjected to ICS analysis. The frequencies of IFNγ^+^ CD3^+^ T cells, IL-17^+^ CD3^+^ T cells, IL-2^+^ CD3^+^ T cells and TNFα^+^ CD3^+^ T cells were quantified. **(E)** Crude PB samples derived from MDA-MB-231 tumor-bearing Hu-CD34^+^ mice (n=3) were subjected to ICS analysis in the absence or presence of catalase (1000 U/ml). The frequencies of IFNγ^+^ CD3^+^ T cells and IL-17^+^ CD3^+^ T cells were quantified. **(F)** A proposed model: in crude tissue samples ICS analysis, the T cell activation agent PMA simultaneously stimulates neutrophils to release H_2_O_2_/ROS, which kills cytokine-expressing T cells, leading to an artifactual result of T cell immunosuppression. Values of n represent biologically independent animals. Data are mean ± s.e.m. *P* values were calculated using one-way ANOVA **(A, B, D)** or unpaired *t*-test **(E)**. **P < 0.01; ***P < 0.001; ****P < 0.0001. NS, not significant.

In the past decade, cancer immunotherapeutic targeting T cell immunosuppression has emerged as one of the most promising treatments in managing a diversity of human cancer types (29). As neutrophils are the most abundant immune cells in humans (30), our findings from the mouse models led us to raise a concern that the artifactual immunosuppression readouts from ICS analysis of human patient specimens could potentially mislead the development of effective immunotherapies. To understand whether neutrophil-associated ICS artifact also arises in analysis of human immune cells, we exploited the humanized NSG™-SGM3 mice (NSG mice expressing human stem cell factor, granulocyte macrophage colony-stimulating factor and IL-3) in which human CD34^+^ hematopoietic stem/progenitor cells had been engrafted and developed into functional human immune system including human neutrophils and T cells (31). Upon a successful xenograft of human MDA-MB-231 breast tumor cells, human neutrophils and T cells were isolated. Consistent with the mouse system, human neutrophils were able to release H_2_O_2_ after stimulation by PMA but not ionomycin (**Figure 5A**). By ICS analysis of human T cells co-cultured with human neutrophils, a prominent neutrophil-mediated suppression of both IFNγ^+^ and IL-17^+^ T cells was found in both spleen and lung samples (**Figure 5B**). This suppression was also identified to occur in other human cytokines including IL-2 and TNFα, which was largely reversed by addition of catalase (**Figures 5C, D**). Collectively, these results suggested that the ICS-associated artifact could similarly exist in human immune cell analysis.

ICS analysis of the peripheral blood (PB) samples is a common test method in evaluation of the immune responses in human patients with cancer, infection and inflammatory diseases (32–34). In our mouse model of 4T1 breast cancer, PB manifests the same artifact (tumor-induced T cell suppression) when assessing IFNγ^+^ CD8^+^ T cells by ICS, while such an “immunosuppression” was not concluded using the IFNγ-eYFP reporter mice by living cell immunostaining and flow cytometric analysis (**Figure S5B**). Using the humanized mice, we found that addition of catalase in the PB samples significantly increased the percentages of IFNγ^+^ and IL-17^+^ human T cells as detected by ICS, suggesting a possible reaction between human neutrophils and human T cells during the sample activation stage in ICS (**Figure 5E**). Thus, special attention should be paid when PMA-involved ICS method is used in patients’ blood sample analyses for T cell responses in clinical studies as PMA induces robust ROS production and cytotoxicity of human neutrophils according to our results and others’ studies (35, 36).

## Discussion

In this study, we defined a previously unrecognized issue with ICS, a widely used cytokine-determining method for T and other immune cells. When the ICS samples contain neutrophils, the T cell activation agent PMA also stimulates neutrophils, which in turn release H_2_O_2_ and kill cytokine-expressing T cells. This causes a robust but artifactual T cell immunosuppression (**Figure 5F**). Although other approaches such as qPCR, enzyme-linked immunosorbent spot (ELISpot) assay, and ELISA can be utilized to detect cytokine production in immune cells, ICS remains the most prevalent tool to determine the intracellular cytokines in individual cells isolated from tissue samples. Based on our results, we therefore recommend conducting neutrophil depletion or T cell purification in ICS analysis, and including complementary methods in measurement of cellular cytokine levels in experimental and clinical blood and tissue samples containing neutrophils. Besides those, other T cell activation methods than using PMA, such as anti-CD3 plus anti-CD28 antibodies and antigen-specific T cell activation, which do not stimulate neutrophils, also avoided the PMA-associated artifact (**Figure S6**). Thus, our improvements to the ICS protocol and proposed alternative methods would benefit a wide array of basic and clinical research.

Such ICS artifact and its resulting false positive result of T cell immunosuppression has the potential to greatly misguide cancer immunology and other immunological research. Cancer-related inflammation is well known to elicit both host neutrophilia and T cell immunosuppression in a wide variety of cancer types (9, 37, 38). This ICS-associated artifact could lead to overinterpretation of T cell immunosuppression caused by cancer-associated inflammation in both preclinical models and clinical sample analyses. Further, T cell immunosuppression is a fundamental target of cancer immunotherapy (39, 40), and an artificial immunosuppression readout could potentially hinder the development of effective immunotherapeutics. In other immunological research such as the emerging COVID-19 studies, blood samples have been routinely used for immune profiling (41, 42), and host neutrophilia and T cell immunosuppression were both reported in severe COVID-19 patients (43, 44). Thus, our results highlight that careful attention should be paid in studies using COVID-19 patient blood samples for ICS analysis.

## Data Availability Statement

The original contributions presented in the study are included in the article/**Supplementary Material**. Further inquiries can be directed to the corresponding author.

## Ethics Statement

The animal study was reviewed and approved by Institutional Animal Care and Use Committee at The Jackson Laboratory.

## Author Contributions

GR, ZG, and QL conceived the project, designed the study and performed the data analysis. ZG and QL performed the *in vivo* and *in vitro* studies, statistical analysis and generated the figures. JS did animal work and mouse colony management. GR, ZG, and QL interpreted the data and wrote the manuscript. All authors contributed to the article and approved the submitted version.

## Funding

This work was supported by grants from the National Institutes of Health (R00-CA188093, R37-CA237307, R01CA251433 and P30-CA034196 to GR). QL was supported by the Pyewacket Fund at The Jackson Laboratory.

## Conflict of Interest

The authors declare that the research was conducted in the absence of any commercial or financial relationships that could be construed as a potential conflict of interest.

## Publisher’s Note

All claims expressed in this article are solely those of the authors and do not necessarily represent those of their affiliated organizations, or those of the publisher, the editors and the reviewers. Any product that may be evaluated in this article, or claim that may be made by its manufacturer, is not guaranteed or endorsed by the publisher.
