# Supplementary material for: An Artifact in Intracellular Cytokine Staining for Studying T Cell Responses and Its Alleviation
Source: Front Immunol. 2022 Jan 21;13:759188. doi: 10.3389/fimmu.2022.759188 (PMC8813780; doi:10.3389/fimmu.2022.759188)
Supplement: Supplementary file 1 [file DataSheet_1.pdf]

Supplementary Information

An Artifact in Intracellular Cytokine Staining for Studying T Cell Responses and Its Alleviation

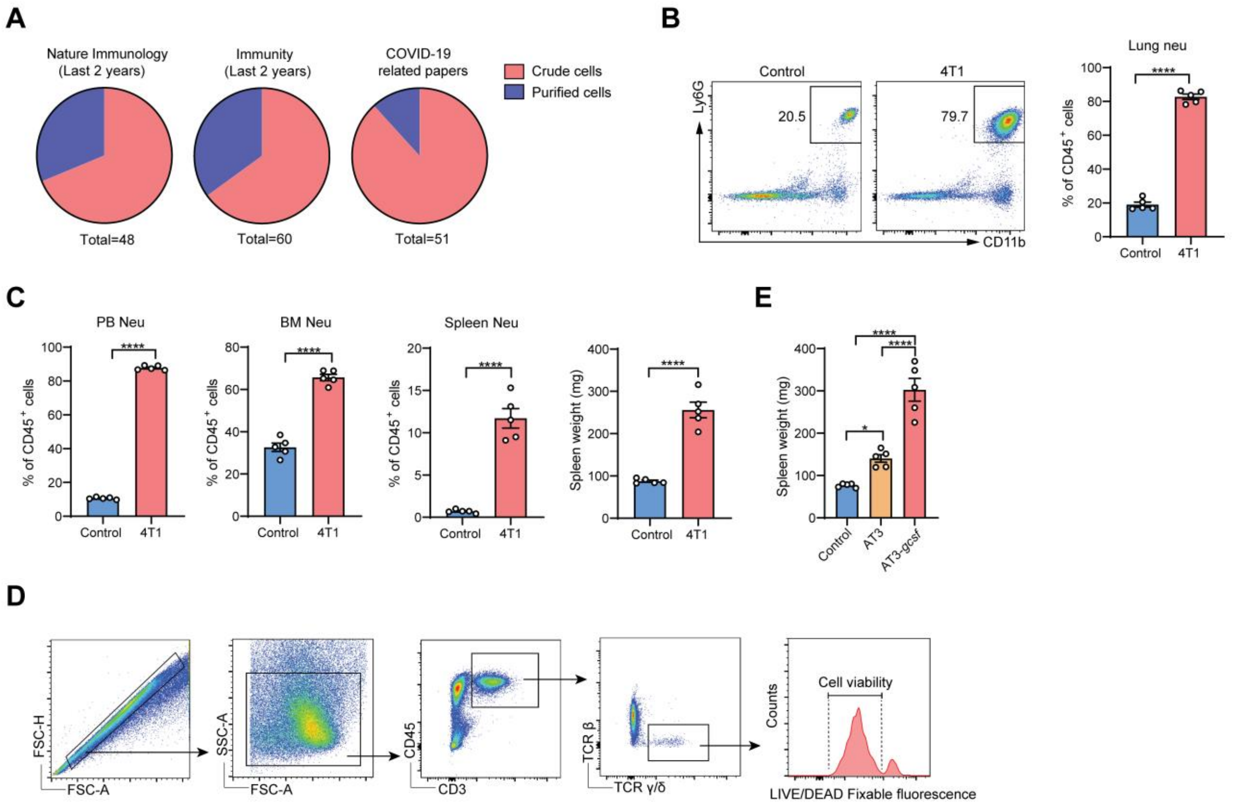

Figure S1. Host neutrophilia in mouse models of breast cancer.

A, Literature analysis of the use of crude cells or purified cells for ICS analysis in the immunology journals “Nature Immunology” and “Immunity” in years 2019-2020 (*left* and *middle*), and in recent COVID-19 related studies (*right*).

B-C, The percentages of neutrophils ($CD45^+CD11b^+Ly6G^+$) in lung (**B**), peripheral blood (PB), bone marrow (BM) and spleen (**C**) in control and 4T1 tumor-bearing mice. Spleen weight, an indicator of inflammation, is also shown in (**C**). n=5 mice per group.

D, The gating strategy for the measurement of T cell viability in ICS analysis.

E, The spleen weights were compared among control, AT3- and AT3-*gcsf* tumor-bearing mice. n=5 mice per group.

Values of n represent biologically independent animals. Data are mean ± s.e.m. *P* values were calculated using unpaired *t*-test (**B-C**) or one-way ANOVA (**E**). *$P < 0.05$; ****$P < 0.0001$.

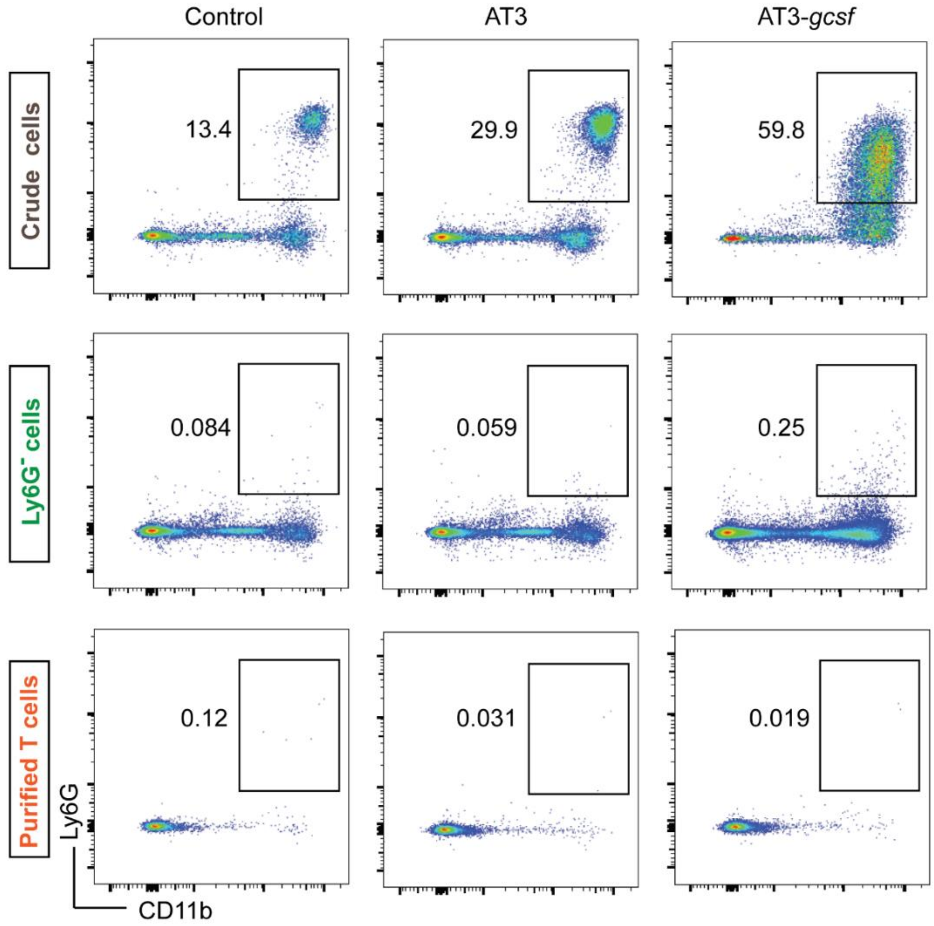

Figure S2. Detection of neutrophil depletion efficiencies in the AT3/AT3-*gcsf* models.

The percentages of $CD11b^{+}Ly6G^{+}$ neutrophils in crude lung samples, lung samples depleted of neutrophils and purified lung T cell samples prepared from naïve control, AT3 tumor-bearing and AT3-*gcsf* mice, were measured by flow cytometry.

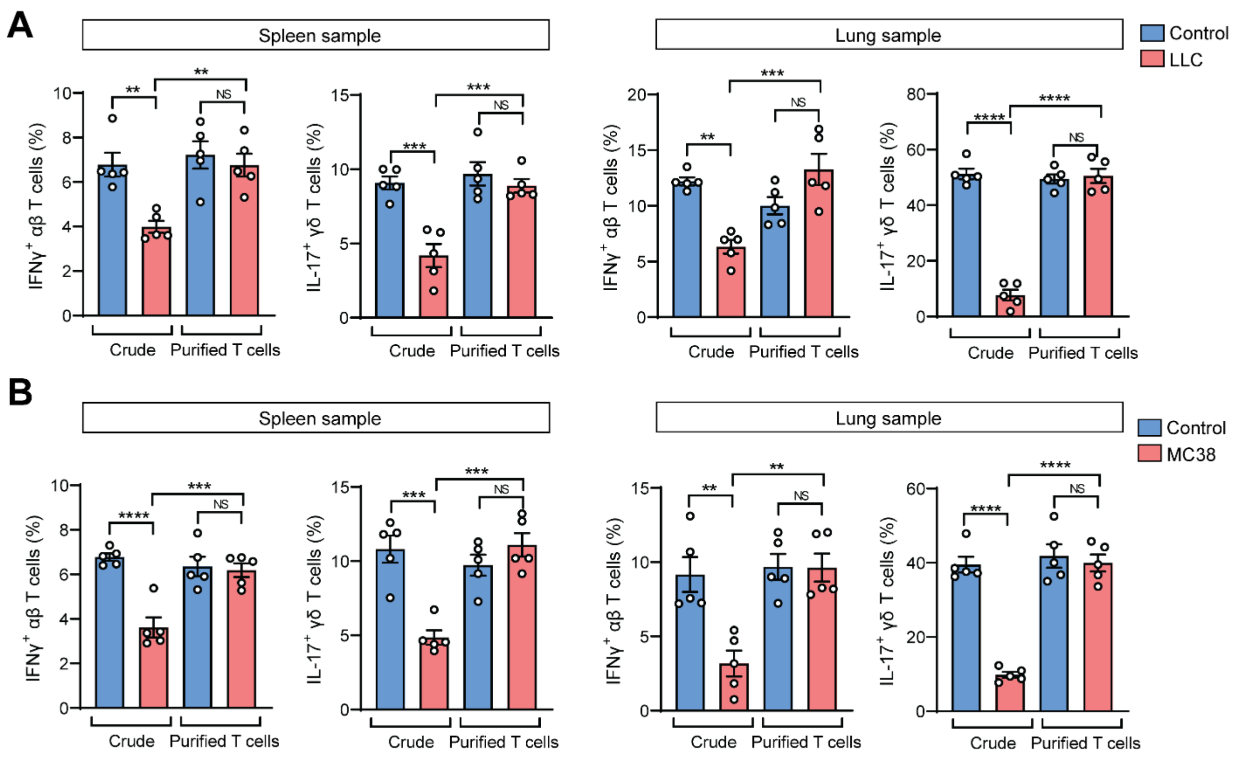

Figure S3. The neutrophil-induced artifact exists in mouse LLC and MC38 tumor models.

The frequencies of IFNγ+ αβ T cells and IL-17+ γδ T cells were determined by ICS in crude spleen or lung samples, or in purified T cell samples prepared from control mice or tumor bearing mice (**A**, LLC model; **B**, MC38 model; n=5 mice per group).

Values of n represent biologically independent animals. Data are mean ± s.e.m. *P* values were calculated using one-way ANOVA. $^{**}P < 0.01$; $^{***}P < 0.001$; $^{****}P < 0.0001$. NS, not significant.

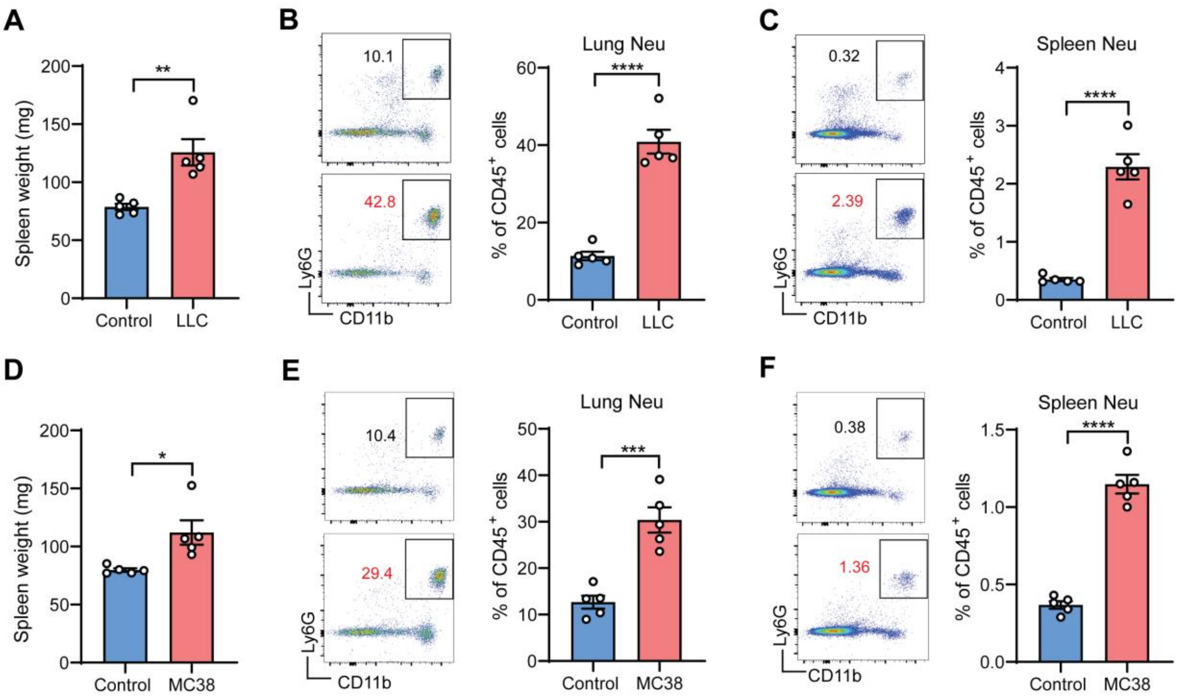

Figure S4. Host neutrophilia in mouse LLC and MC38 tumor models.

A-F, Spleen weights, an indicator of inflammation, were compared between control and tumor-bearing mice (**A**, LLC model; **D**, MC38 model; n=5 mice per group). The percentages of neutrophils ($CD45^+CD11b^+Ly6G^+$) in lungs and spleen of control mice or tumor bearing mice (**B-C,** LLC model; **E-F**, MC38 model; n=5 mice per group) were measured by flow cytometry.

Values of n represent biologically independent animals. Data are mean ± s.e.m. P values were calculated using unpaired t-test. *$P < 0.05$; **$P < 0.01$; ***$P < 0.001$; ****$P < 0.0001$.

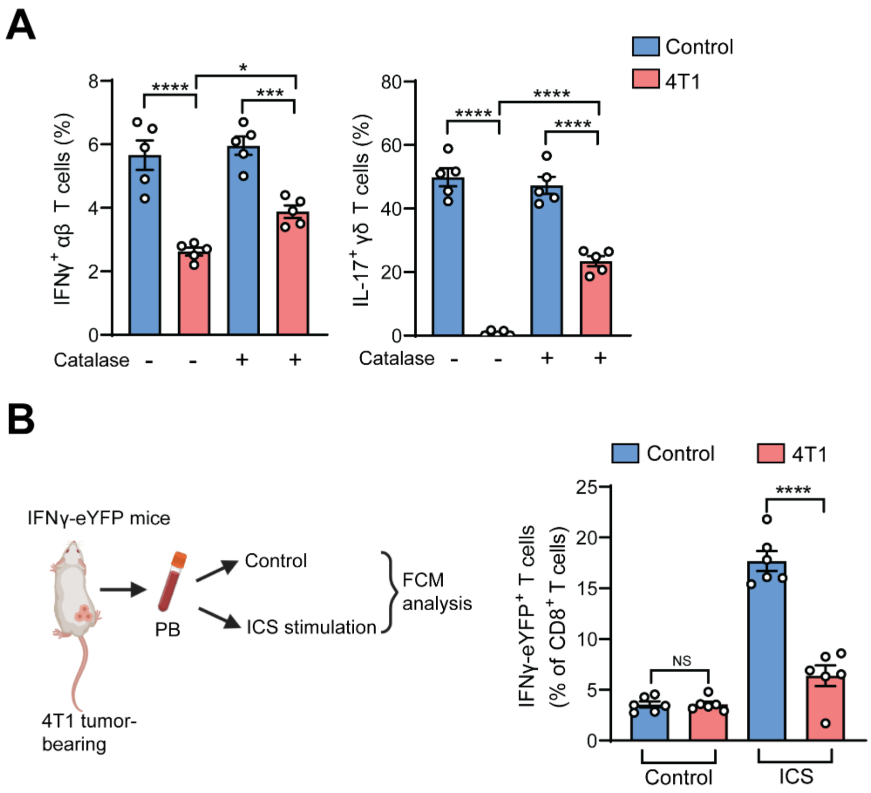

Figure S5. Addition of catalase partially resolved the ICS-associated artifact.

A, The frequencies of IFNγ$^+$ αβ T cells (*left*) and IL-17$^+$ γδ T cells (*right*) in crude lung samples from naïve control or 4T1 tumor-bearing mice (n=5 per group) were measured by ICS analysis with or without addition of catalase (1000 U/ml).

B, The frequencies of IFNγ-eYFP$^+$ CD8$^+$ T cells in PB samples were measured by living cell immunostaining with or without ICS stimulation (25 ng/ml PMA and 1 µg/ml ionomycin) for 4 hours, followed with flow cytometry (FCM) analysis. n=6 mice per group.

Values of n represent biologically independent animals. Data are mean ± s.e.m. *P* values were calculated using one-way ANOVA. **P* < 0.05; ****P* < 0.001; *****P* < 0.0001. NS, not significant.

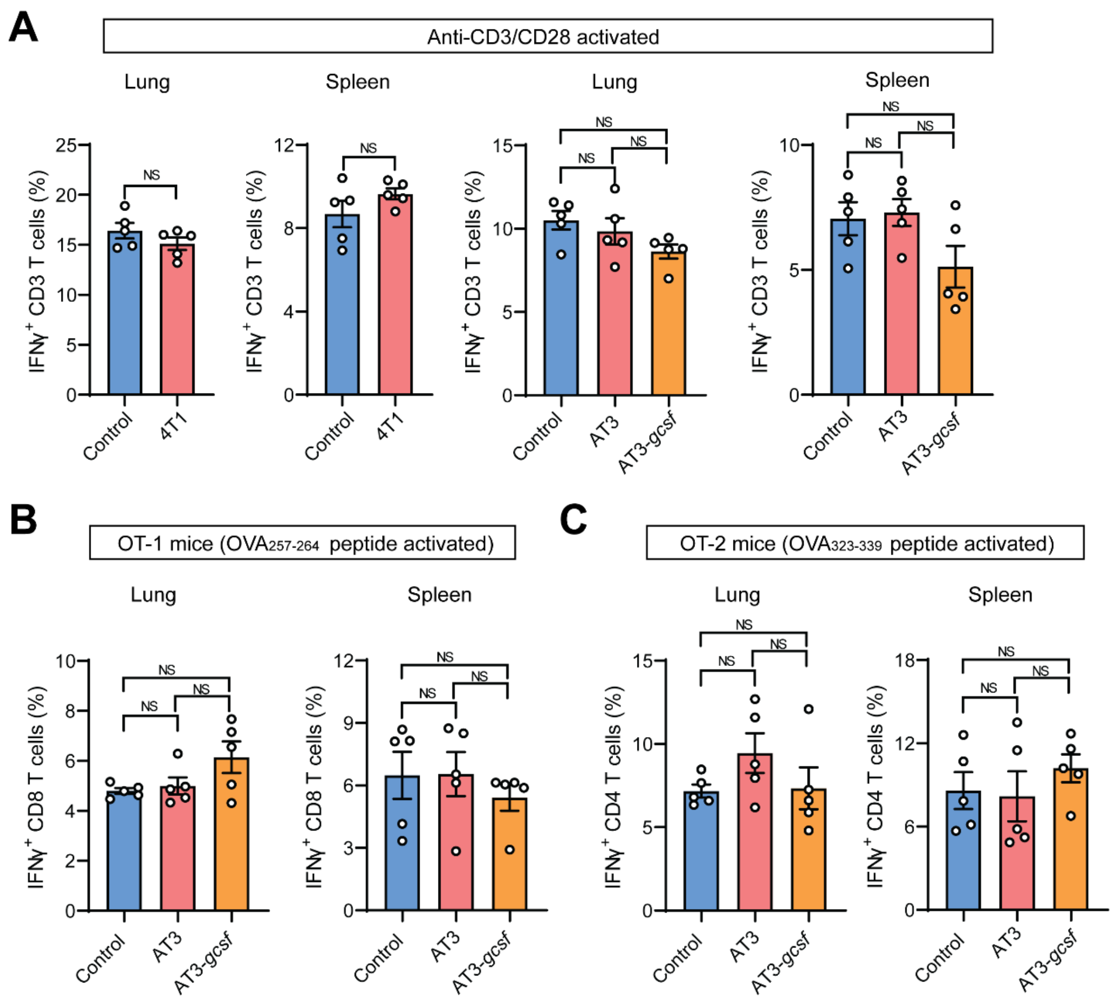

Figure S6. Activation of T cells by anti-CD3/anti-CD28 or the specific antigen avoids PMA-associated artifact.

Crude lung or spleen samples were prepared from the indicated orthotopic 4T1- or AT3/AT3-*gcsf* tumor bearing mice (n=5 per group). (**A**) The samples were activated by plate-bound anti-CD3 (5 µg/ml) and soluble anti-CD28 (1 µg/ml) for 24 hours, and the frequencies of IFNγ$^+$ CD3$^+$ T cells in total CD3$^+$ T cells were measured by ICS analysis. (**B**) The samples were activated by $OVA_{257-264}$ peptide (10 µg/ml) for 24 hours, and the frequencies of IFNγ$^+$ CD8$^+$ T cells in total CD8$^+$ T cells were measured by ICS analysis. (**C**) The samples were activated by $OVA_{323-339}$ peptide (10 µg/ml) for 24 hours, and the frequencies of IFNγ$^+$ CD4$^+$ T cells in total CD4$^+$ T cells were measured by ICS analysis.

Values of n represent biologically independent animals. Data are mean ± s.e.m. *P* values were calculated using unpaired *t*-test (two groups) or one-way ANOVA (more than 2 groups). NS, not significant.
